# Supplementary material for: Causal Relationship between Aging and Anorexia Nervosa: A White-Matter-Microstructure-Mediated Mendelian Randomization Analysis
Source: Biomedicines. 2024 Aug 16;12(8):1874. doi: 10.3390/biomedicines12081874 (PMC11351342; doi:10.3390/biomedicines12081874)
Supplement: Supplementary file 1 [file biomedicines-12-01874-s001.zip › Supplementary Figure/Figure S8/Leaveoneout plot of MR analysis for TL to ubm-b-1529.pdf]

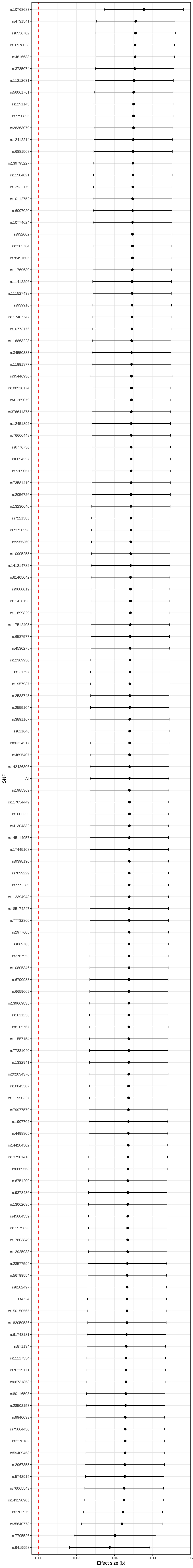
rs10768683
rs4731541
rs6536702
rs16978028
rs4616688
rs3785074
rs11212631
rs56061761
rs1291143
rs7790856
rs28363070
rs12412214
rs6881568
rs139795227
rs11584821
rs12932179
rs10112752
rs6007020
rs10774624
rs932002
rs2282764
rs78491606
rs11769630
rs11412296
rs111527438
rs939916
rs117407747
rs10773176
rs116863223
rs34550383
rs11991877
rs35446936
rs188918174
rs41269079
rs376641875
rs12451892
rs76666449
rs6776756
rs6054257
rs7209057
rs73581419
rs2056726
rs13230646
rs7221585
rs73730598
rs9955360
rs10905255
rs141214782
rs61405042
rs9600019
rs11426156
rs11699829
rs117512405
rs6587577
rs4530278
rs12369950
rs131797
rs1957937
rs2538745
rs2555104
rs3891167
rs611646
rs80324517
rs4695407
rs142426306
All
rs1985369
rs117034449
rs1003322
rs41304832
rs145114957
rs17445108
rs9398196
rs7099229
rs7772289
rs112394943
rs185174247
rs77732866
rs2977608
rs869785
rs3767952
rs10805346
rs6790988
rs6659669
rs139669835
rs1611236
rs8105767
rs11557154
rs77231040
rs1332941
rs202034370
rs10845387
rs111950327
rs79977579
rs1907702
rs4498805
rs144204502
rs137901416
rs6669563
rs6751209
rs9878436
rs13062095
rs45604339
rs11579626
rs17803849
rs12925933
rs28577594
rs56799554
rs8102497
rs4724
rs150150565
rs182059586
rs61748181
rs871134
rs11117354
rs76219171
rs66731853
rs80116508
rs28502153
rs9940099
rs75664430
rs2276182
rs59409453
rs2967355
rs57742915
rs76065543
rs143190905
rs2763979
rs35640778
rs7705526
rs9419958
SNP
0.00
0.03
0.06
0.09
0.12
Effect size (b)
